# A case report of endoscopic resection for the treatment of duodenal Brunner's gland adenoma with upper gastrointestinal bleeding

**DOI:** 10.1097/MD.0000000000023047

**Published:** 2020-12-24

**Authors:** Ma Liang, Zhang Liwen, Song Jianguo, Dai Juan, Shen Ting, Chen Jianping

**Affiliations:** a Department of Digestive Disease, The First People's Hospital of Changzhou, The Third Affiliated Hospital of Soochow University; b Department of Pediatrics, the Second People's Hospital of Changzhou, Affiliate Hospital of NanJing medical University, Changzhou, Jiangsu; c Department of Gastroenterology, The People's Hospital of Wuqia, Xin Jiang, China.

**Keywords:** Brunner's gland adenoma, diagnosis, gastrointestinal bleeding, treatment

## Abstract

**Rationale:**

Gastrointestinal bleeding as the first sign of Brunner's gland adenoma (BGA) is an extremely rare, and its clinical features and treatment methods have not been well described.

**Patient concerns:**

We described a 81-year-old female patient with coronary artery disease and chronic atrial fibrillation presenting with presenting with gastrointestinal bleeding requiring blood transfusion.

**Diagnoses:**

The diagnosis of our case mainly refered to radiologic imaging and endoscopic examination. Histological result was compatible with BGA.

**Interventions:**

This mass lesion (6 × 7 cm diameter) was successfully totally removed by endoscopic submucosal dissection (ESD) for more than three hours.

**Outcomes:**

The patient was followed up for 6 months to date without recurrence.

**Lessons:**

Endoscopic removal is considered as a safe and low-risk treatment for elderly patients with severe underlying diseases presenting with gastrointestinal bleeding.

## 1 Introduction

Brunner's gland adenoma (BGA), also named as ploypoid hamartoma and Brunneroma, is a extremely rare benign duodenal tumor arising from the Brunner's gland of the duodenum.^[1]^ They were first described as a benign hamartomatous lesion characterized by the proliferation of Brunner's glands in 1835 by a American anatomist, Cruveilhier.^[2]^ Generally, they are asymptomatic and discovered by chance during upper gastrointestinal endoscopy or on an upper gastrointestinal series, as they are rarely larger than 2 cm. Occasionally, some present with chronic abdominal pain, nausea, vomiting, and anemia when they may be larger than 5 cm, and located in the duodenum.^[3]^ When they reach giant dimensions, they may obstruct the gastric outlet or duodenum, which requires surgery or endoscopic resection.^[4]^ Although a few case reports have previously described BGA patients with gastrointestinal bleedings,^[5–6]^ there are no systematic reports on the clinical features and treatments.

In this case report, we retrospectively presented a rare patient with a duodenal polypoid mass of more than 7 cm who was admitted to our department with upper gastrointestinal bleeding and on which endoscopic mucosal resection was performed. Furthermore, we also systematically reviewed the patients clinical presentation, imaging features, endoscopic picture, and possible treatment.

## 2 Case presentation

**Figure 1:**
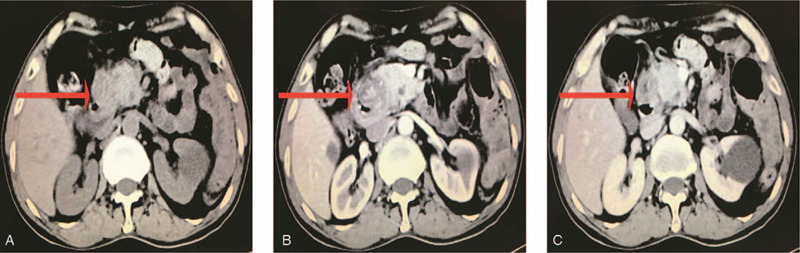
Conventional and enhanced CT images. Large (4.5 × 5.5 cm diameter) hypervascular exophytic mass in the Duodenum in CT (red arrow head).

**Figure 2:**
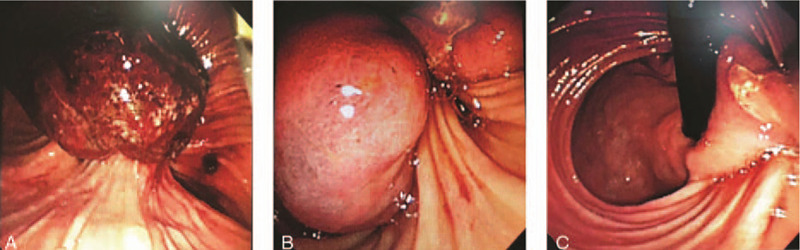
Endoscopic appearance. Endoscopy showed a large pe-dunculated mass (A/B) located on the posterior surface of duodenal bulb with stigmata of recent hemorrhage (C).

**Figure 3:**
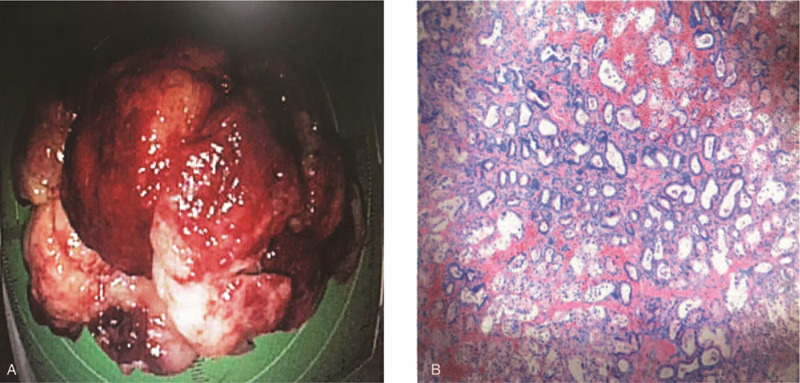
Histological examination. The mass in the resected specimen had stigmata of recent hemorrhage, 6 × 7 cm in size. (B) Microscopic examination showed extensive proliferation of Brunner's glands.

A 81-year-old female patient experienced with the chief complaints of melena for 1 weeks, accompanied by a progressive epigastric pain, weakness and fatigue, was recruited at the inpatient service of Department of Gastroenterology, the First People's Hospital of Changzhou. She had a past medical history significant for coronary artery disease and chronic atrial fibrillation for more than 10 years subsided by anticoagulants. She had chronic episodic upper central abdominal pain for many years subsided by antisecretory medications; however, no history of melena previously. Presently, she denied any recent use of non-steroidal anti-inflammatory drugs (NSAIDS). Upon admission, her vital signs were quite unstable: heart rate was 80 beats/minutes, blood pressure was 102/70 mm Hg; and respiratory rate was 20 breaths/minutes. Physical examination revealed an anemic man in weakened condition. Hematological evaluation revealed severe anemia with a hemoglobin level of 66 g/L. In addition, as listed in Table 1, other laboratory data, including coagulation function, liver function, and serum tumor markers, all within normal limits. The patient received a total of 2 transfusions of erythrocyte concentrates, after which the hemoglobin level remained stable at 80 g/L. The chest and abdomen CT showed a large (4.5 × 5.5 cm diameter) hypervascular exophytic mass, which appeared to originate from the descending duodenum (Fig. 1A-C). Furthermore, there were no signs of invasion or dissemination. The emergency upper gastrointestinal endoscopy (EGD) revealed a large pedunculated mass located on the posterior surface of duodenal bulb with stigmata of recent hemorrhage (Fig. 2A-C). Endoscopic ultrasonography (EUS) revealed a submucosal polypoid mass, which was not possible to see the entirety of the mass due to its large size and moving stem. There was no evidence of Helicobacter pylori-associated gastritis. Furthermore, biopsies were not conclusive as the mass showed a quick inclination to bleed during biopsies. After multidisciplinary discussions, this mass lesion was considered to be most likely to be BGA, followed by gastrointestinal stromal cell tumor (GIST). Given the patients advanced age, heart disease and the risk of bleeding at any time, endoscopic resection was planned. After the patient signed the consent, this mass lesion (6 × 7 cm diameter) was successfully totally removed by endoscopic submucosal dissection (ESD) for more than three hours (Fig. 3A). The postoperative period was uneventful and the pathologic diagnosis was assessed as Brunner's gland adenoma (Fig. 3B). Her tarry stools stopped after endoscopic therapy, and her hemoglobin levels improved to 90 g/L after multiple blood transfusions. The follow-up EGD after 6 months showed white scar change on the posterior surface of the duodenal bulb.

**Table 1:** Laboratory observation upon admission.

Characteristics	Index	Normal range
Blood		
WBC (^∗^10^9^ /L)	7.01	4.0–10.0
RBC (^∗^10^12^ /L)	2.55	3.5–5.5
HB (g/L)	73–66-	120–155
PLT (^∗^10 9 /L)	278	100-300
Coagulation function		
PT (s)	10.8	9.0–13.0
APTT (s)	25.6	19.0–34.5
Liver function		
ALT (u/L)	33	9–50
AST (u/L)	62	10–45
γ-GT (u/L)	23	10–60
ALP (u/L)	121	40–125
TP (g/L)	53.2	60–82
ALB (g/L)	30.5	35–55
CHE (u/L)	4542	3000–8000
Serum tumor markers		
AFP (ng/mL)	2.01	0–8
CEA (ng/mL)	0.91	0–5
CA199 (U/mL)	5.38	0–37
CA125 (U/mL)	7.25	0–35

## 3 Ethic statement

Our institutional review board was waived due to the retrospective nature of the study. Written informed consent was obtained from the patients parents for the publication of this case report.

## 4 Discussion

Brunner's gland, firstly described in 1688, are submucosal glands that secrete mucus with an alkaline PH, thereby aiding in the neutralization of the acidic chyme and facilitating the intestinal digestion.^[7]^ BGA is an extremely rare benign tumor accounting less than 10% of all benign duodenal tumors. They mostly occur in the bulb (57%), but also occur in the descending (27%) and horizontal (5%) parts of duodenum.^[8]^ Furthermore, the frequency of occurrence of BGA the pyloric canal, jejunum, and proximal ileum is less than 5%. Although these are mostly smaller than 2 cm, they are also found to be larger than 2 cm, even reaching a size of 12–13 cm.^[9]^

The exact etiology of the BGA is currently unknown. However, there are many postulations regarding the pathophysiology of BGA. It is generally accepted that hyperacidic conditions could stimulate the formation of Brunner's gland hyperplasia. Some studies have reported a possible association between BGA and hyperchlorhydria in patients with chronic gastric erosions and duodenal ulcers.^[10]^ However, some scholars have recently opposed this view because they found that antacid treatment could not alleviate this lesion.^[11]^ Moreover, chronic Helicobacter pylori (HP) infection might play a major role in the process of BGA formation. Some reports have observed the coexistence between HP infection and BGA in 71% of cases.^[12]^ However, others did not find a clear correlation.^[3]^ This difference might be attributed to the high prevalence of HP infection. Furthermore, the excessive local irritation from vagal stimuli and unidentified antral hormones might also be the cause.^[10]^ Our patient has history of antiacid medication and HP testing was negative. Therefore, further studies on the pathophysiology are needed to be carried out.

Although BGA have been found in patients range under 1 year to 80 years, they are usually observed in middle-aged patients.^[13]^ In addition, BGA did not show differences in gender and race distribution.^[13]^ Most of BGA patients are usually asymptomatic and found by accident during esophagogastroduodenoscopy or imaging studies. A few cases show nonspecific gastrointestinal symptoms, such as nausea, bloating, or vague abdominal pain.^[3]^ Furthermore, when the BGA lesions are larger in size, there are rare clinical manifestations incuding gastrointestinal obstruction pancreatitis and obstructive jaundice.^[4,14]^ Our case presented with BGA-related blood loss, which led to hemodynamic instability. Although some studies have reported cases with gastrointestinal bleeding as the main clinical manifestation,^[5–6]^ our case show larger lesion size. In addition, age of the patient was older (81 years) than previously reported of 76 years old.^[15]^

The diagnosis of BGA relies on radiologic imaging and endoscopic examination.^[16–17]^ Computed tomography is more sensitive for identifying the absence of extra-luminal extension of BGA. In addition, a barium swallow can show filling defects in the area of pathology. However, radiologic imaging can not distinguish benign and malignant lesions, nor can it be distinguished from other submucosal lesions such as leiomyomas, lipomas, and lymphomas.^[16]^ Endoscopy examination can demonstrate the specific location and appearance of BGA. Furthermore, endoscopic ultrasound (EUS) can identify the origin, extent and vascularity of these submucosal lesion. Endoscopic biopsies are usually negative or show only non-specific Brunner's glandular hyperplasia due to insufficient tissue. In our case, histological biopsy could not be done due to the bleeding susceptibility of the lesion.^[17]^ Therefore, the diagnosis of our case mainly refered to radiologic imaging and endoscopic examination. The main differential diagnoses are carcinoid, lymphoma, gastrointestinal stromal tumor, PeutzJeghers polyps, prolapsed pyloric mucosa, or aberrant pancreatic tissue.^[7,9,14]^

The standard treatment method for BGA has not been established. The current main treatment options are different according to size, symptoms and suspicious of malignancy. Some people suggested conservative treatment for asymptomatic small BGA because it was considered to have no neoplastic potential and only needed follow-up.^[3]^ However, others advocated endoscopic resection in order to prevent complications.^[4]^ For symptomatic and larger BGA leading to gastrointestinal obstruction or bleeding, they usually needed endoscopic or surgical treatment.^[4,16–17]^ When possible, endoscopic polypectomy or mucosal resection was advised, while for larger tumors or endoscopic interventions fail, the surgical resection should be carefully considered, bearing in mind the higher risk of complications.^[17]^ In our case, considering the patients advanced age, heart disease, and risk of complications (bleeding and perforation), we successfully removed the lesion by endoscopic mucosal dissection (ESD). At the 6-month follow-up, the patient was doing well and no signs of bleeding.

## 5 Conclusions

In summary, we reported a extremely rare case of BGA patient with gastrointestinal bleeding as the main clinical manifestation. The lesion was diagnosed by radiologic imaging and endoscopic examination. ESD could be a safe and low-risk treatment for elderly patients with severe underlying diseases. However, because our observation is limited to 1 patient, more patients and longer follow-up are necessary.

## Author contributions

**Conceptualization:** Chen Jianping.

**Formal analysis:** Dai Juan.

**Project administration:** Song Jianguo.

**Resources:** Shen Ting.

**Writing – original draft:** Ma Liang.

**Writing – review & editing:** Zhang Liwen.
